# Support-Material-Free Microfluidics on an Electrochemical Sensors Platform by Aerosol Jet Printing

**DOI:** 10.3390/s19081842

**Published:** 2019-04-18

**Authors:** Nicolò Giuseppe Di Novo, Edoardo Cantù, Sarah Tonello, Emilio Sardini, Mauro Serpelloni

**Affiliations:** 1 Laboratory of Bio-Inspired & Graphene Nanomechanics, Department of Civil, Environmental and Mechanical Engineering, University of Trento, Via Mesiano, 77, 38123 Trento, Italy; 2 Department of Information Engineering, University of Brescia, Via Branze 38, 25123 Brescia, Italy; e.cantu@unibs.it (E.C.); s.tonello@unibs.it (S.T.); emilio.sardini@unibs.it (E.S.)

**Keywords:** voltammetric sensors, Aerosol Jet Printing, glucose sensing, 3-D printing, support-material-free microfluidics

## Abstract

Printed electronics have led to new possibilities in the detection and quantification of a wide range of molecules important for medical, biotechnological, and environmental fields. The integration with microfluidics is often adopted to avoid hand-deposition of little volumes of reagents and samples on miniaturized electrodes that strongly depend on operator’s skills. Here we report design, fabrication and test of an easy-to-use electrochemical sensor platform with microfluidics entirely realized with Aerosol Jet Printing (AJP). We printed a six-electrochemical-sensors platform with AJP and we explored the possibility to aerosol jet print directly on it a microfluidic structure without any support material. Thus, the sacrificial material removal and/or the assembly with sensors steps are avoided. The repeatability observed when printing both conductive and ultraviolet (UV)-curable polymer inks can be supported from the values of relative standard deviation of maximum 5% for thickness and 9% for line width. We designed the whole microfluidic platform to make the sample deposition (20 μL) independent from the operator. To validate the platform, we quantified glucose at different concentrations using a standard enzyme-mediated procedure. Both mediator and enzyme were directly aerosol jet printed on working electrodes (WEs), thus the proposed platform is entirely fabricated by AJP and ready to use. The chronoamperometric tests show limit of detection (LOD) = 2.4 mM and sensitivity = 2.2 ± 0.08 µA/mM confirming the effectiveness of mediator and enzyme directly aerosol jet printed to provide sensing in a clinically relevant range (3–10 mM). The average relative standard inter-platform deviation is about 8%. AJP technique can be used for fabricating a ready-to-use microfluidic device that does not need further processing after fabrication, but is promptly available for electrochemical sample analysis.

## 1. Introduction

The sensitive quantification of specific biomolecules and analytes in biological fluids, drinkable water, or food has a key role in medicine, biotechnological, and environmental research [1,2]. The presence of specific biomolecules or ions above their normal levels in human fluids affects biochemical cycles and causes adverse health effects [3]. For example, diabetes is a chronic disease [4] and it is reflected by blood glucose concentrations higher or lower than the normal range (4.4–6.6 mM) [5]. Early diabetes detection can help to reduce the risk of serious complications [6,7]. Besides, proteins can also give important information about patient health and predict a pathology insurgence in time [8,9]. Similarly, the possibility to detect contaminants, specific ions, or metals in drinkable water, beverages, or food, when still in low concentration, might bring a significant improvement in terms of food waste and of effects on human wealth [10,11]. Several highly specific and sensitive analytical techniques have been used to detect target analytes in biological or environmental samples such as enzyme-linked immunosorbent assay (ELISA) [12], surface plasmon resonance (SPR) [13], surface-enhanced Raman scattering (SERS) [14] and high-performance liquid chromatography (HPLC) [15]. The disadvantages are the high costs and the need for expensive equipment and trained personnel [3]. In recent years, the demand for disposable biosensors with high sensitivity, low limit of detection (LOD), repeatability, miniaturization, and cost efficiency has received increasing attention for early diagnosis and health monitoring [16]. Electrochemical biosensors (EB) are good candidates for scalable production of point-of-care (PoC) disposable devices [3,17,18]. They are a feasible solution for analyzing the content of a biological fluid sample by directly converting a biological event into an electronic signal [19]. 

Recently, printed electronics have been increasingly investigated as a convenient and promising strategy to achieve miniaturization, low cost, and ease of surface modification of EB [20,21]. Presently, the printing techniques adopted most frequently for these applications are screen printing (SP) and ink-jet printing (IJP). They both allow resolution up to 50–100 µm, required to provide proper geometrical properties of electrochemical sensors for a wide range of biotechnological applications such as chemical detection, DNA, or protein recognition [22,23]. Low-cost examples are the paper-based microfluidic devices (μPAD) that perform an electrochemical [24,25,26] or a colorimetric [27,28] assay, and the multi-working electrodes screen-printed electrochemical sensors commercialized by Dropsens [29]. Even though planar three-electrode electrochemical configurations are the most commonly adopted for printed EB, they show some practical problems: (i) the functionalization and sample droplets are commonly deposited by hand using a micropipette. In the presence of small electrodes (e.g., lower than 1 mm) and low amount of fluid (few μL), deposition is difficult and strongly depends on the operator, introducing errors and high variability; (ii) the lack of an effective physical barrier that separates working electrodes (WE) and counter electrodes (CE) can cause an improper functionalization of the CE and distort the analysis; and (iii) in specific methods of analysis, the sample has to be split, transported, and collected on functionalized electrodes. In these cases, the fluid could be not properly split, transported, or collected, and the sensors can overestimate or underestimate the real analyte concentration. 

Focusing on the requirements of precise control of fluids, low reagent consumption, and parallel multi-analysis, the integration of biosensors with a proper microfluidics environment represents a valuable strategy, that has been intensely investigated in recent literature [1,3,16,17,24,27,28,30,31]. Thus far, the traditional technique adopted for microfluidic circuit fabrication is poly-dimethylsiloxane (PDMS)-based soft lithography. Despite the fact that it provides high quality and allows the fabrication of high-complexity structures [17,31], it requires several slow and expensive steps to get to the final product, from mask production and PDMS lithography to the assembly with sensors. To overcome these issues, Additive Manufacturing (AM) methods represent a powerful tool to combine electrodes with a suitable 3D environment for biological assays [32]. AM techniques have been proposed as valid alternatives with improved resolution, flexibility, range of materials, ease of processing, and reduced price [30]. Furthermore, direct microfluidic printing on pre-existing sensors would decrease discarded pieces, as it would avoid assembly and allow a faster and automated production. Besides, current AM techniques still have limitations in that sense. Stereolithography (SLA) [33] presents practical difficulties in the non-solidified resin removal inside microchannels and needs assembly with sensors. IJP can print low-viscosity ink and therefore requires support material [34,35] to produce microchannels; its removal can affect the surface and electrical properties of micro/nano-structured electrodes. Fused Deposition Modeling (FDM) can realize channels without sacrificial material but it has limitations in the XY resolution, the feature minimum size, and the channel wall roughness [36,37].

Among AM techniques, Aerosol Jet Printing (AJP) has been investigated in previous works as promising to improve resolution, high-performance and miniaturized electrical components [38]. As demonstrated in [21], AJP electrodes show that higher resolution, lower LOD, and improved repeatability could be obtained compared to SP. 

In this paper, considering the discussed issues both in terms of electrodes miniaturization and microfluidic fabrication, we report the design, fabrication, and test of an easy-to-use electrochemical sensor platform with microfluidics entirely realized with AJP. To the best of our knowledge, it is the first example of aerosol jet microfluidic printing directly on electronics without any support material by photopolymer Norland Optical Adhesive 81 (NOA 81) jetting and UV curing. AJP microfluidics does not require assembly steps, it is relatively fast, it minimizes the structural material amount, it can also be printed on non-planar surfaces, and it opens new possibilities of integration with miniaturized electronics. In this specific application, we realized a triangular section microchannel with a base 220 μm wide but considering the AJP resolution [39] and the NOA 81 UV-curing results, even channels in the order of tens of microns could be investigated. To study AJP microfluidic printing capabilities, we realized a platform composed of six EB in a hexagonal shape integrated with a customized NOA 81 structure with six microchannels that branch off from a central inlet to each WE chamber. We chose the number six as many examples in the literature have this order of magnitude; multi-analysis devices with 2, 3, 4, or 6 WEs are reported in [25,26,27,28,29,30] depending on the application. With AJP, it is possible to adapt the geometry to the requirements very freely and the only additional cost is the time taken to draw in Computer-Aided Design (CAD). Validation has been performed quantifying glucose using a standard enzyme-mediated procedure, directly printing both mediator and enzyme using AJP technique. The microfluidic platform allows improvement and control of the sample deposition on WEs, lowering operator dependency and ensuring a proper deposition of reduced sample volumes. The miniaturization of the electrochemical sensors ensures reduction of electrochemical noise, voltage drop, and maximization of analyte detection [40]. Finally, the direct AJP of mediator and enzyme makes the device standardized and ready to use. 

## 2. Materials and Methods 

### 2.1. Platform Design and Material Choice

**Figure 1:**
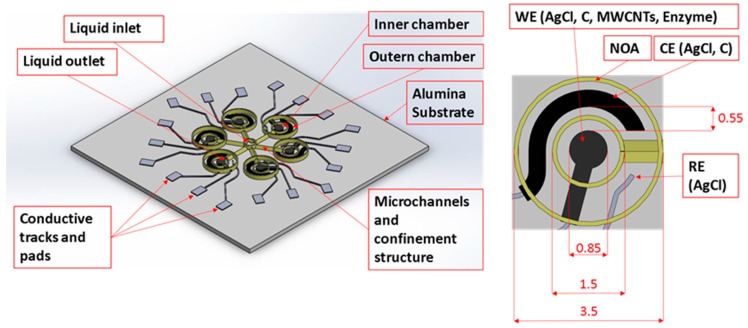
Schematic representation of the final prototype.

The schematic representation of the designed microfluidic sensor platform is reported in Fig. 1; each layer and material used in the development of the platform is indicated. Alumina is the selected substrate material due to its mechanical properties, good adhesion because of its porosity, and ease of handling. We purchased the substrates in 22 × 22 mm squares. The platform is composed of six electrochemical cells with a diameter of 3.5 mm, each of them characterized by three electrodes; WE is designed in AutoCAD with 0.85 mm diameter; the nominal distance between WE and CE is 0.55 mm. A UV-cured material to manage liquid samples surrounds the whole structure. The yellow central structure in Figure 1 permits the delivery of the sample on WEs by introducing a micropipette in the inlet and injecting. The six bigger chambers serve to contain the buffer solution. We design microfluidics to contain 20 μL of sample fluid including the amount that remains in the channels and inlet. Inner chambers have a diameter of 1.5 mm and the channel length is 2.8 mm. The CAD geometry of the channel is an isosceles triangle with base angle of 50° and base length equal to 300 μm. The central inlet nominal diameter is about 1.1 mm and it is dimensioned with interference on a 1.1 mm tip micropipette.

**Figure 2:**
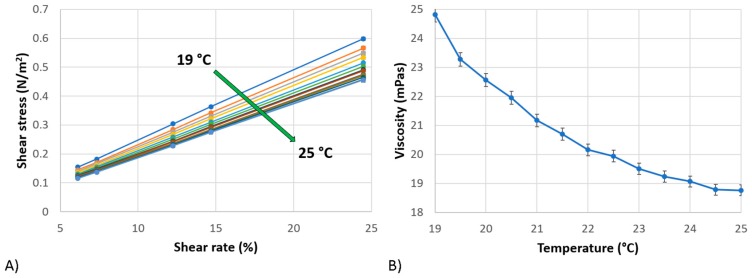
(**A**) Shear stress vs Shear rate, showing a complete linear behavior for temperature in the range 19–25 °C for AgCl ink; (**B**) Viscosity as a function of temperature for AgCl ink.

The materials employed for EB are silver chloride (AgCl) for conductive tracks, pads, and Reference Electrode (RE), and carbon (C) ink for WE and CE. Furthermore, multi-wall carbon nanotube (MWCNT) ink was selected and printed over WE to enhance both electrical performance and the surface-to-volume ratio for biofunctionalization. Silver chloride ink (XA-3773) was purchased by Fujikura Kasei Co., Ltd. (Shibakouen Minato-ku, Tokyo, Japan) together with its own thinner. The ink was chosen with Ag/AgCl weight proportion ratio of 8/2. A dilution of the ink, with its specific thinner, was mandatory to obtain a proper viscosity for the printing stage (ink starting viscosity was 300 ± 50 dPa∙s), following the equations reported in the literature regarding a two-component blend [41]. Rotational viscosity measurements were performed using Viscotech VR 3000 MYR Viscometers modelV2-L (C/Lleida, 17-23 · Pol. Ind. L’empalme, 43712 Llorenç del Penedès, Tarragona, Spain) to measure the viscosity after dilution. The tests were performed in a common range of ambient temperatures, from 19 °C to 25 °C, evaluating the behavior of the ink for each 0.5 °C in the abovementioned range. Different rotating speeds were selected: 5, 6, 10, 12, and 20 rpm. Figure 2A shows the linear behavior of AgCl ink for different testing temperatures, demonstrating that it can be considered to be a Newtonian fluid in measurement range. Temperature has an important role in the printability of the material, as it is clearly visible in Figure 2B: the higher the temperature, the lower the viscosity. In conclusion, the ink was deposited at 23 °C with a viscosity of about 19.5 mPa∙s.

Carbon ink (EXP 2652-28) was acquired from Creative Materials Inc. (Ayer, MA, USA), characterized by a starting viscosity of 250 mPa∙s. Nink 1000, commercialized by NANOLAB (Waltham, MA, USA), is the abovementioned MWCNTs ink: it has a viscosity about 3 mPa∙s and it contains carboxyl (COOH) functionalized carbon nanotubes in an aqueous suspension (the viscosity is proximal to the one of water) with the minimum concentration of additives to impart long-term stability and printability to the ink. UV-curable polymer NOA 81 was purchased by Norland Products (Cranbury, NJ, USA). We selected NOA 81 for several reasons: fast curing with a 365 nm light, high viscosity (300 mPa∙s at 25 °C) that limits the polymer spread before curing [42], transparency, natural hydrophilicity [43] to permit a spontaneous capillary flow inside channels, low shrinkage during curing because it is a crosslinking process without evaporation, biocompatibility with biomolecules and cells [44,45], good mechanical properties to have a robust structure with minimum material employed, excellent adhesion on a wide range of material, and chemical resistance [46].

### 2.2. Fabrication Process

**Figure 3:**
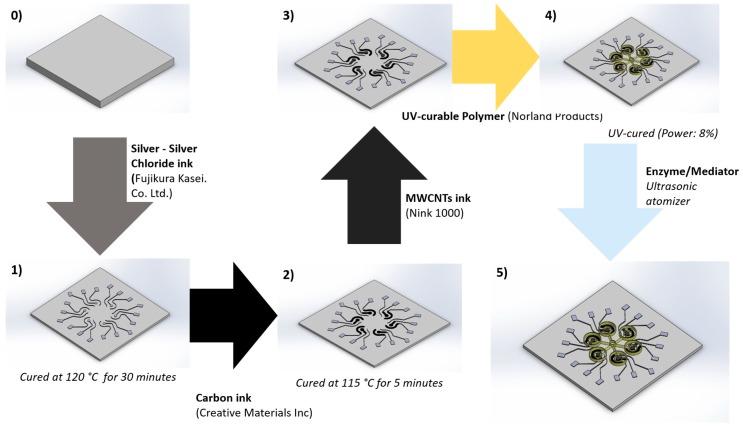
AJP process for microfluidic sensors platform fabrication.

AJ300 system commercialized by Optomec was used to fabricate and functionalize the electrochemical sensors platform with microfluidics. Table 1 summarizes the AJP process parameters for each ink and Figure 3 shows the process scheme. 

**Figure 4:**
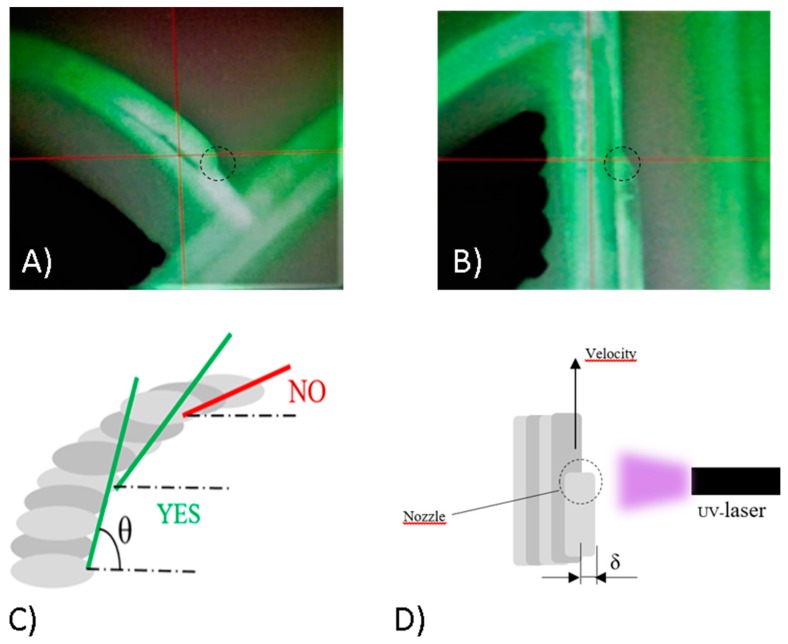
Process camera photo during printing of (**A**) a chamber wall and (**B**) a microchannel cantilevered line; representation of the critical angle (**C**); printing scheme and line offset δ (**D**).

AgCl, C, and MWCNT inks were printed with two consecutive depositions, followed by their own specific heat treatments, using a 200 μm nozzle tip. Pneumatic atomization was selected. NOA 81 printing was performed in a single deposition step and UV curing is performed during the printing process. The UV Curing System is the Light Emitting Diode (LED) Spot-type Panasonic ANUJ6180 series, model 6423 (Panasonic, Kadoma, Osaka, Japan) characterized by a spot diameter of 3 mm, wavelength of 365 nm in correspondence of the peak and a peak intensity of 17200 mW∙cm^−2^ at the distance of 8 mm. The selected power was 8% of the peak intensity. NOA 81 printability requires particular attention. The AJP pneumatic atomizer produces a mist of micro-droplets dispersed inside nitrogen (atomizer flow). The virtual impactor permits the reduction and filtering of the mist removing the smaller droplets by controlling a negative pressure (exhaust flow). The remaining flow (aerosol flow) is accelerated and collimated in the nozzle through a coaxial nitrogen flow (sheath flow). The ratio between sheath and aerosol flows (χ) requires fine tuning to reduce overspray. Indeed, as precisely explained in [47], bigger droplets deviate to the center of the flow more than the smaller ones due to Saffman forces. Thus, the droplet size distribution along the jet section changes. In the center of the jet, the presence of larger particles that, after impact, form the core of the printed line increases, whereas on the sides there are smaller droplets that form overspray. As discussed in [48], higher values of χ reduces this effect but implies also less material deposition and can cause nozzle clogging. In printing NOA 81, the printed line requirements are thickness in the order of tens of microns to lower the printing time, no porosity, and as little overspray as possible. NOA 81 overspray increases with temperature and it is probably related to the atomizer droplet size distribution change due to viscosity and surface tension lowering. With optimized printing parameters (see Table 1), NOA droplets coalesce during the collimation and is possible to generate a dense NOA jet with a low amount of overspray. The UV laser quickly solidifies the jet as it hits the substrate before it can spread and flow (Figure 4A). Stacked cantilevered solid lines can be printed to create an overhang wall without any sacrificial material (Figure 4B).

Therefore, microchannels can be fabricated without any support material. The idea is to generate and print 2D drawings obtained by longitudinal slicing of the CAD channel at different heights. There is not yet software dedicated to AJP that allows the making of slices from a virtual 3D object and converting them directly into machine files. We used Solidworks for 3D modeling, Nettfabb to create slices of the desired thickness and AutoCAD VMTools provided by Optomec to generate machine files. The layer thickness (*h*) is chosen in accordance with the NOA line thickness. Therefore, a preliminary geometrical analysis was performed with Filmetrics Profilm 3D optical profilometer (Filmetrics Inc., 10655 Roselle St., San Diego, CA, USA). With the parameter reported in Table 1, the measured thickness for one single line was about 25 μm and about 10–20% less for cantilevered or stacked lines. The analysis showed a slight lowering of thickness for cantilevered or stacked lines is because the new line adapts to the real geometry of the previous one. Therefore, we chose *h* equal to 20 μm. The channel transversal section geometry that minimizes the layer number necessary to close the channel is the triangular one with the lowest angle possible (θ) between the wall and the substrate. Reasonably, θ has a minimum value θcr below which collapse can occur before solidification (Figure 4C). Indeed, when the thickness of the layers is fixed, if the angle decreases, the offset δ between the cantilevered lines increases. Higher offset means minor support area for the new line (Figure 4D). We have found that θcr is approximately 45° for our printing parameters. However, fixing the jet flow rate and increasing the printing velocity, the line thickness decreases, thinner CAD slices can be generated, and therefore it is possible to have a smaller offset between lines also for θ < θcr. Thus, also quasi-circular channels could be investigated. Considering these geometrical constraints, it is possible to model and print microfluidics in one step with a great variety of shapes including inlets, outlets, and chambers.

**Figure 5:**
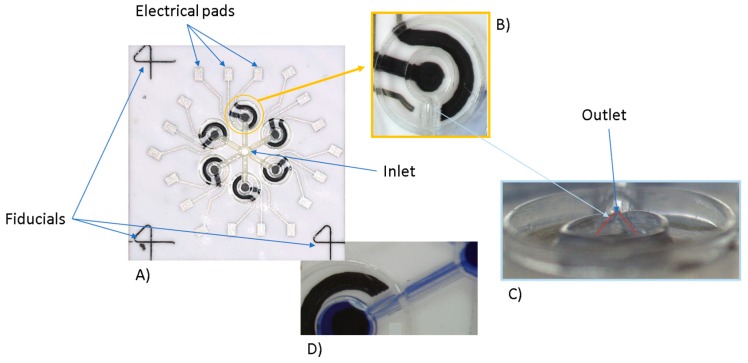
Final platform produced by means of AJP (**A**), with specific details of the two concentric limiting circles (**B**) and a frontal view of the triangular channel (**C**); a channel filled with pen ink (**D**).

The final platform with microchannel and chamber details is shown in Figure 5. In this figure, the prototype is shown with its specific geometrical and production features (Figure 5A), together with magnification regarding a single chamber (Figure 5B) and the profile of the triangular channel (Figure 5C). It is also possible to observe the inlet and the outlet of the microfluidic system developed and a channel filled with pen ink to test its correct usage (respectively Figure 5A,C,D). 

After completing the overall platform, both in terms of sensors and of microfluidic channels, AJP has been finally adopted to functionalize WEs with specific chemicals required to perform glucose sensing. In detail, first, a ferro/ferri-cyanide (Fe^2+^/Fe^3+^) 5 mM solution has been printed only on the WEs, to act as a mediator during chemical reaction for glucose sensing. Furthermore, a solution of 300 U/mL of Glucose Oxidase (GOD) in Acetate Buffer (pH 5) has been printed to provide ready-to-use sensors. Both the solution of mediator and enzyme have been printed via ultrasonic atomization (UA). Despite that fact that we focused on glucose sensing, the proposed approach can be used with different measurement methods and applications with other enzymes or chemicals. 

### 2.3. Geometrical Analysis and Electrical Resistances 

Geometrical and electrical tests were performed on printed lines. Filmetrics Profilm 3D optical profilometer (Filmetrics Inc., 10655 Roselle St., San Diego, CA, USA) was used to evaluate the thickness of the printed lines. It is based on state-of-the-art white-light interferometry (WLI), a non-contact optical method for surface height measurement on 3-D structures, to measure surface profiles and roughness down to 0.05 µm. The instrument works in the range of 50 nm–10 mm with substrates and materials characterized by a reflectance from 0.05–100 %. The system implements a 5MP camera, the Nikon CF IC Epi Plan 20x model (field-of-view: 1.0 × 0.85 mm).

To measure the width of the microfluidic structure in which the liquid sample would be inflated during glucose sensing, an optical microscope by Orma Scientific NB50T (trinocular zoom 0.8x–5x–LED), with its devoted software and HDMI MDH5 camera model, was used to acquire the images and to evaluate the features of the printed elements (Orma Scientific, Sesto San Giovanni, Milan, Italy). 

Electrical resistance was evaluated using the digital bench-top multimeter Hewlett–Packard 34401a (HP, Palo Alto, CA, USA), applying testing probes to the extremities of each path, in standardized and repeatable points, thus measuring the resistance offered by all its length. Each measure has been repeated ten times, to ensure the proper calculation of the mean values and of the standard deviations. Resistivity was then calculated from the classical equation R = ρ∙l∙S-1 where R is resistance, ρ is resistivity, l is the length of the considered path and S its section. 

### 2.4. Electrochemical Analysis 

The electro-active surface area (A_real_) was evaluated for every electrode-type from Randles-Sevcik equation (1) for reversible reaction, as well described in [22], by performing Cyclic Voltammetry (CV) at 0.1 V/s scan rate (ν) in a phosphate buffer saline (PBS) (50 mM, pH 7.0) containing a 5 mM concentration (C) of ferro/ferri-cyanide ([Fe(CN)6]^3−/4−^). Indeed, the electrochemical couple Fe^2+^/Fe^3+^ redox process has a very well-known diffusion coefficient (D = 6.20 × 10^−6^ cm^2^).

(1) $$I{\;}_{\mathrm{rev}}\;=\;\pm 0.446\;{\mathrm{nFA}}_{\mathrm{real}}C\;\sqrt{\frac{\mathrm{nFD}\nu }{RT}},$$

Furthermore, an electrochemical characterization was performed to investigate the effect of scan rate on oxidation and reduction currents and potentials. In detail, multiple CVs in the presence of 5 mM [Fe(CN)6]^3−/4−^ were performed at different scan rates (25, 50, 100, 150, and 200 mV/s) in the potential range −0.2 to 0.4 V using the commercially available portable potentiostat Palmsens3 EIS (Palmsens, Compact Electrochemical Interfaces, Houten, Utrecht, The Netherlands).

### 2.5. Glucose Sensing

**Figure 6:**
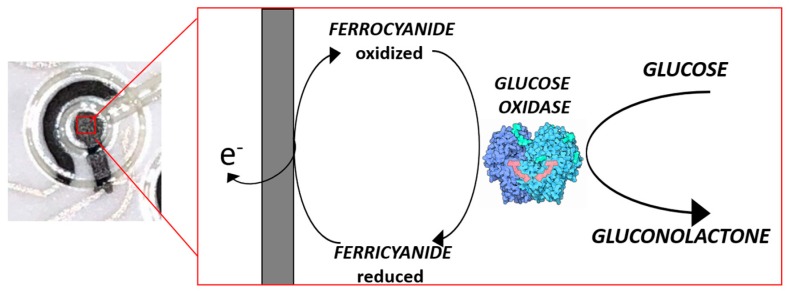
Mechanism of enzyme-mediated glucose sensing.

Standard solutions of D-Glucose in Deionized (DI) water have been prepared with the following concentration: 0 mM, 5 mM, 10 mM, 25 mM, 50 mM, 100 mM. For each test, the platforms have been directly used after printing. 20 μL of D-Glucose solution was injected using a micropipette from the central inlet, thus to provide the solution only to the enzyme-coated WEs, to allow the following chemical reactions in presence of GOD (2), producing an amount of electrons proportional to the concentration of glucose, then transported to the electrodes thanks to the mediator (M) (3–4) (Figure 6):

Glucose + GOD(ox) → Gluconic acid + GOD(red),(2)

GOD(red) + 2M(ox) → GOD(ox) + 2 M(red) + 2H^+^, (3)

2M(red) → 2M(ox) + 2e^−^, (4)

Five seconds after loading the sample, 10 μL of a buffer solution of DI water containing 50 mM of PBS as supporting electrolyte (pH 7.0) was dropped onto each electrochemical cell and a potential of +500 mV vs. Ag/AgCl was applied. Chronoamperometric measurements were recorded for 60 s with the abovementioned potentiostat. Current value at 60 s was taken as output to compare the different concentrations. Three microfluidic platforms have been tested for each concentration.

## 3. Results and Discussion

### 3.1. Geometrical Analysis and Electrical Resistances

**Figure 7:**
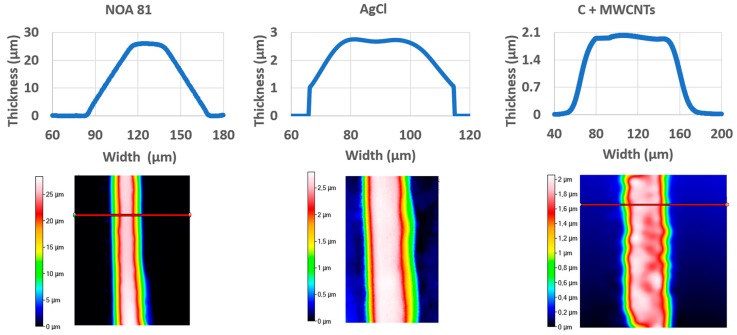
Profiles obtained thanks to Filmetrics 3D optical profilometer for NOA 81, carbon with MWCNTs and AgCl.

The measured values of thicknesses, widths, and sections are reported in Table 2. The geometrical data obtained are in agreement with our previous work [21], presenting a better relative standard deviation, denoting an improvement thanks to dedicated process parameters. Figure 7 presents the profiles measured with the optical profilometer. NOA 81 channels’ width presents an average value of 211 μm (relative standard deviation is about 7.8%).

Results from electrical tests show resistivity data in agreement with the nominal values of the manufacturers, considering the specific process parameters for each ink. The use of the thinner to achieve the proper final viscosity has affected AgCl experimental resistivity value (89.5 × 10^−8^ Ω∙m) that is higher compared with the nominal one reported by Fujikura Kasei. Co. Ltd. (56 × 10^−8^ Ω∙m). Finally, C experimental resistivity (7.7 × 10^−4^ Ω∙m) was slightly decreased compared to the one given by Creative Materials (25 × 10^−4^ Ω∙m), due to the choice performed during the heat treatment in terms of duration and temperature and to multiple material deposition.

### 3.2. Electrochemical Analysis

The electrochemical analysis performed using at 100 mV/s allowed calculation from the Randles-Sevcik equation described in the method section the average active area of each platform as 18.92 ± 1.05 cm^2^, confirming a high reproducibility of the electrochemical cells geometry and of the active area available for experiments. These are in agreement with what was demonstrated in previous works [21], were AJP was shown to ensure a lower variability when compared to SP.

**Figure 8:**
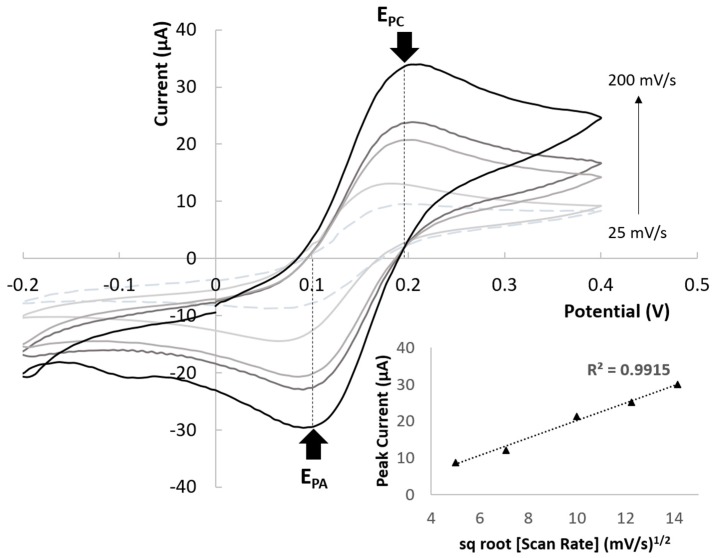
Electrochemical characterization of AJP sensors in presence of 5mM Ferri-ferrocyanide solution. Cyclic voltammetries at increasing scan rates (25, 50, 100, 150, and 200 mV/s) and linear relation between peak current height and the square root of the scan rate confirming the diffusion control of the reaction.

Figure 8 shows CV plots for a single electrochemical AJP cell in presence of 5 mM [Fe(CN)6]^3−/4−^ with increasing scan rates of 25, 50, 100, 150, and 200 mV/s. Both the oxidation and reduction peak currents linearly increased with the square root of scan rate, which indicates that the redox reaction is diffusion controlled [49,50]. Furthermore, the ratio between the anodic and cathodic peak current is near to unity for each scan rate. Regarding the cathodic and anodic potential (Epc and Epa), they both appear almost perfectly independent from the scan rate. Both the previous findings suggest a reversible behavior of the overall known reversible redox systems such as the ferri/ferrocyanide ones [51]. 

### 3.3. Glucose Sensing

**Figure 9:**
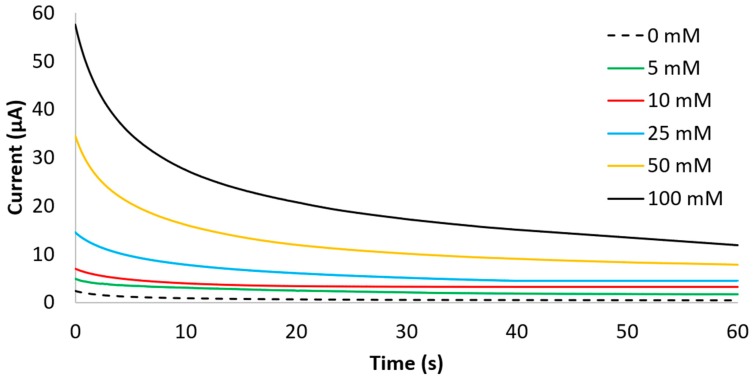
Chronoamperograms at +500 mV recorded at different glucose concentration.

Chronoamperograms obtained with different glucose concentrations suggested the possibility to correlate the increase in the steady state current at 60 s with the increase of glucose concentration (Figure 9). The trend of glucose calibration plot appears to be logarithmic over the full range of concentration (0–100 mM). This suggests a higher sensibility of the overall system for lower concentrations and a saturation for higher glucose concentrations.

**Figure 10:**
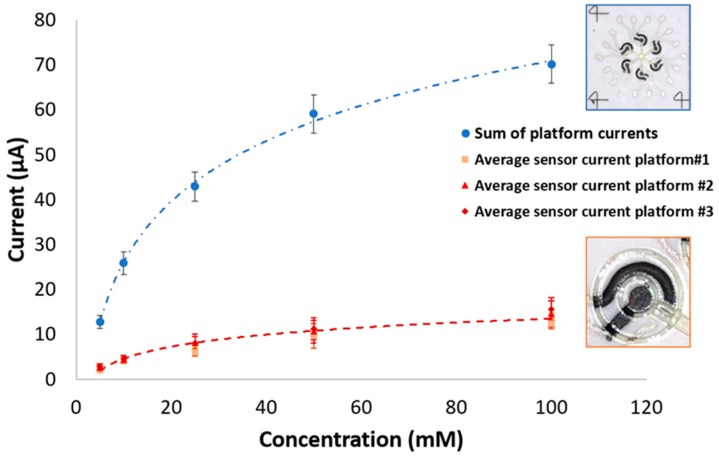
Current values at 60 s (blank corrected) at different glucose concentrations: comparison between single sensor’s average value on each repetition and averaged sum of the currents in a single platform.

The platform output that estimates glucose concentration of a 20 μL sample is the current sum. Figure 10 shows the average of the three platform output values obtained as sum of the six currents of each platform for each concentration, blank corrected. The sum values are compared to the values of the average currents inside each platform for each concentration. The outputs of the proposed platform permit correct discrimination between every level of concentration, and at the lowest values. The LOD, calculated using the 3-sigma rule, taking as reference the blank standard deviation, is 2.4 mM. This value appears to be suitable for monitoring glucose concentrations typical of human blood, usually included in the range between 3 mM and 10 mM [52]. Furthermore, due to the versatility of the AJP procedure, and the possibility of easily improving the number of layers, these results could be optimized by varying the amount of mediator and enzyme on WEs.

Looking at the relative standard deviations of the measurements summarized in Table 3, an average variability of about 15% can be observed when averaging the currents from six sensors of the same platform. This intra-platform variability (15%) suggests a proper functioning of the microfluidics in homogeneously distributing the sample among the six different measurements points. The average of the relative standard deviations of current sums is about 8%. This inter-platform variability (8%) suggests a high reliability of the overall printing strategy with a proper control of all the different steps involved for the final production of the platform. The values obtained for the relative standard deviation appear in agreement with previous works performed fabricating AJP sensors for protein detection [21]. Furthermore, despite the fact that the intention of our paper was not the optimization of the LOD performance in term of glucose sensing, used as a mere validation of the AJP platform, our results appear to agree in terms of sensitivity with the results of optimized screen-printed sensors using the very same enzymatic mechanism for glucose detection [53]. Specifically, the sensitivity obtained with our platform in the range 0–10 mM was equal to 2.2 ± 0.08 µA/mM, in agreement with that obtained in [53] of 2.13 ± 0.06 µA/mM.

## 4. Conclusions

A fully AJP electrochemical microfluidic sensing platform has been designed, fabricated, and tested. The variability observed when printing both conductive and UV-curable polymer inks was evaluated from the values of relative standard deviation lower than 5% for thickness and 9% for line width. This geometrical deviation suggests the potential of AJP technique for realizing sensors for accurate and repeatable environmental and clinical sample analysis. The AJP electrochemical microfluidic sensing platform has been validated by performing a standard enzyme-mediated procedure for glucose sensing. The average relative intra-platform and inter-platform standard deviations observed from the current average evaluation (15% and 8%, respectively) suggested the possibility of guiding the positioning of the sample on miniaturized electrodes, to replicate the same analysis on separate platforms and, in future developments, to perform multiple analysis. This provides an improvement in term of lower operator dependency, reduction of sample waste, and of analysis variability. Results from glucose sensing (LOD = 2.4 mM and sensitivity = 2.2 ± 0.08 µA/mM) confirmed the effectiveness of mediator and enzyme direct AJP to provide sensing in a clinically relevant range (3–10 mM). This suggests the usefulness of this technique for providing a ready-to-use device that does not need further processing after fabrication, but is promptly available for electrochemical sample analysis. In future works, we will test different other methods of analysis and multi-analysis capabilities by differently functionalizing the electrodes.

## Figures and Tables

**Table 1:** Printing process parameters.

Process Parameters	AgCl	C	NOA 81	Nink 1000	Mediator/Enzyme (UA)
Sheath gas flow (SCCM)	55	40	40	50	60
Atomizer flow (SCCM)	750	805	1395	670	40
Exhaust flow (SCCM)	720	770	1365	580	/
Process speed (mm·s^−1^)	2	4	1.5	2	2
Plate temperature (°C)	65	70	/	40	/
Current (mA)	/	/	/	/	500

**Table 2:** Thickness and sections of deposited inks.

Material	Thickness (μm)	Relative Standard Deviation (%)	Width (μm)	Relative Standard Deviation (%)	Section (μm^2^)
AgCl	2.71	3	51.8	3.5	136.3
C + MWCNTs	1.97	5	127.8	9	262.74
NOA 81	26.06	1	96.75	4	1580.31

**Table 3:** Intra- and inter-platform variability.

Concent. (mM)	Platform (#)	Average (µA)	St.Dev. (µA)	Relative St. Dev %	Average Sum (µA)	St.Dev. Sum (µA)	Relative St. Dev %
**100.00**	**1**	12.38	1.13	9.13	70.15	4.25	6.06
	**2**	14.60	2.87	19.66			
	**3**	15.60	2.52	16.15			
**50.00**	**4**	10.64	1.40	13.16	59.09	4.26	7.21
	**5**	9.65	0.40	4.15			
	**6**	11.26	2.02	17.94			
**25.00**	**7**	8.13	1.42	17.47	42.93	3.24	7.54
	**8**	8.03	1.87	23.29			
	**9**	6.01	0.86	14.31			
**10.00**	**10**	4.60	0.70	15.22	25.86	2.52	9.70
	**11**	4.32	0.83	19.21			
	**12**	4.02	0.60	14.93			
**5.00**	**13**	2.89	0.60	20.76	12.81	1.42	11.06
	**14**	2.61	0.44	16.86			
	**15**	2.08	0.29	13.94			

## References

[1] Saem S., Zhu Y., Luu H., Moran-Mirabal J. (2017). Bench-top fabrication of an all-PDMS microfluidic electrochemical cell sensor integrating micro/nanostructured electrodes. Sensors.

[2] Hart J., Honeychurch K., Westmacott K., Crew A., Hughes G., Pemberton R. (2016). Recent Advances in the Fabrication and Application of Screen-Printed Electrochemical (Bio)Sensors Based on Carbon Materials for Biomedical, Agri-Food and Environmental Analyses. Biosensors.

[3] Nesakumar N., Kesavan S., Li C.-Z., Alwarappan S. (2019). Microfluidic Electrochemical Devices for Biosensing. J. Anal. Test..

[4] Roglic G. (2014). Global report on diabetes. World Heal. Organ..

[5] Wang J. (2008). Electrochemical glucose biosensors. Electrochem. Sensors Biosens. Their Biomed. Appl..

[6] Hughes G., Pemberton R.M., Nicholas P., Hart J.P. (2018). Fabrication of Miniaturised Screen-printed Glucose Biosensors, Using a Water-based Ink, and the Evaluation of their Electrochemical Behaviour. Electroanalysis.

[7] Nery E.W., Kundys M., Jeleń P.S., Jönsson-Niedziólka M. (2016). Electrochemical glucose sensing: Is there still room for improvement?. Anal. Chem..

[8] So Y.T., Sabbagh M.N., Herbert C., Boxer A., Karydas A., Sparks D.L., Robinson W.H., Takeda-Uchimura Y., Miller B.L., Leszek J. (2007). Classification and prediction of clinical Alzheimer’s diagnosis based on plasma signaling proteins. Nat. Med..

[9] Kim H.J., Li H., Collins J.J., Ingber D.E. (2015). Contributions of microbiome and mechanical deformation to intestinal bacterial overgrowth and inflammation in a human gut-on-a-chip. Proc. Natl. Acad. Sci. USA.

[10] Hayat A., Marty J.L. (2014). Disposable screen printed electrochemical sensors: Tools for environmental monitoring. Sensors.

[11] Meadows D. (1996). Recent developments with biosensing technology and applications in the pharmaceutical industry. Adv. Drug Deliv. Rev..

[12] Lequin R.M. (2005). Enzyme immunoassay (EIA)/enzyme-linked immunosorbent assay (ELISA). Clin. Chem..

[13] Singh P. (2016). SPR Biosensors: Historical Perspectives and Current Challenges. Sensors Actuators B Chem..

[14] Doering W.E., Piotti M.E., Natan M.J., Freeman R.G. (2007). SERS as a foundation for nanoscale, optically detected biological labels. Adv. Mater..

[15] Ma C., Sun Z., Chen C., Zhang L., Zhu S. (2014). Simultaneous separation and determination of fructose, sorbitol, glucose and sucrose in fruits by HPLC-ELSD. Food Chem..

[16] Wu J., Liu Y., Lin F., Rigatto C., Dong M. (2018). Lab-on-chip technology for chronic disease diagnosis. NPJ Digit. Med..

[17] Lee G.H., Lee J.K., Kim J.H., Choi H.S., Kim J., Lee S.H., Lee H.Y. (2017). Single Microfluidic Electrochemical Sensor System for Simultaneous Multi-Pulmonary Hypertension Biomarker Analyses. Sci. Rep..

[18] Riahi R., Shaegh S.A.M., Ghaderi M., Zhang Y.S., Shin S.R., Aleman J., Massa S., Kim D., Dokmeci M.R., Khademhosseini A. (2016). Automated microfluidic platform of bead-based electrochemical immunosensor integrated with bioreactor for continual monitoring of cell secreted biomarkers. Sci. Rep..

[19] Wang J. (2006). Analytical Electrochemistry.

[20] Pasinszki T., Krebsz M., Tung T.T., Losic D. (2017). Carbon nanomaterial based biosensors for non-invasive detection of cancer and disease biomarkers for clinical diagnosis. Sensors.

[21] Cantù E., Tonello S., Abate G., Uberti D., Sardini E., Serpelloni M. (2018). Aerosol Jet Printed 3D Electrochemical Sensors for Protein Detection. Sensors.

[22] Couto R.A.S., Lima J.L.F.C., Quinaz M.B. (2016). Recent developments, characteristics and potential applications of screen-printed electrodes in pharmaceutical and biological analysis. Talanta.

[23] Li J., Rossignol F., Macdonald J. (2015). Inkjet printing for biosensor fabrication: Combining chemistry and technology for advanced manufacturing. Lab Chip.

[24] De Oliveira T.R., Fonseca W.T., de Oliveira Setti G., Faria R.C. (2019). Fast and flexible strategy to produce electrochemical paper-based analytical devices using a craft cutter printer to create wax barrier and screen-printed electrodes. Talanta.

[25] Wang P., Ge L., Yan M., Song X., Ge S., Yu J. (2012). Paper-based three-dimensional electrochemical immunodevice based on multi-walled carbon nanotubes functionalized paper for sensitive point-of-care testing. Biosens. Bioelectron..

[26] Dungchai W., Chailapakul O., Henry C.S. (2009). Electrochemical Detection for Paper-Based Microfluidics. Anal. Chem..

[27] Chiang C.K., Kurniawan A., Kao C.Y., Wang M.J. (2019). Single step and mask-free 3D wax printing of microfluidic paper-based analytical devices for glucose and nitrite assays. Talanta.

[28] Dungchai W., Chailapakul O., Henry C.S. (2010). Use of multiple colorimetric indicators for paper-based microfluidic devices. Anal. Chim. Acta.

[29] Campuzano S., Pingarrón J., Reviejo Á., Pellicanò A., Ruiz-Valdepeñas Montiel V., Cosio M., Torrente-Rodríguez R. (2016). Simultaneous Determination of the Main Peanut Allergens in Foods Using Disposable Amperometric Magnetic Beads-Based Immunosensing Platforms. Chemosensors.

[30] Bohr A., Colombo S., Jensen H. (2018). Future of Microfluidics in Research and in the Market.

[31] Chen J., Zhou Y., Wang D., He F., Rotello V.M., Carter K.R., Watkins J.J., Nugen S.R. (2015). UV-nanoimprint lithography as a tool to develop flexible microfluidic devices for electrochemical detection. Lab Chip.

[32] Ragones H., Schreiber D., Inberg A., Berkh O., Kósa G., Freeman A., Shacham-Diamand Y. (2015). Disposable electrochemical sensor prepared using 3D printing for cell and tissue diagnostics. Sensors Actuators B Chem..

[33] Devarenne S.T.P., Han A., Darwin S., Reyes R., Reyes D.R., Folch A., Minhas H., Gaitan M., Stubbs J., Lee A. (2014). Mail-Order Microfluidics: Evaluation of Stereolithography for the Production of Microfluidic Devices. Lab Chip.

[34] Walczak R., Adamski K. (2015). Inkjet 3D printing of microfluidic structures—On the selection of the printer towards printing your own microfluidic chips. J. Micromech. Microeng..

[35] Alfadhel A., Ouyang J., Mahajan C.G., Forouzandeh F., Cormier D., Borkholder D.A. (2018). Inkjet printed polyethylene glycol as a fugitive ink for the fabrication of flexible microfluidic systems. Mater. Des..

[36] Gaal G., Mendes M., de Almeida T.P., Piazzetta M.H.O., Gobbi Â.L., Riul A., Rodrigues V. (2017). Simplified fabrication of integrated microfluidic devices using fused deposition modeling 3D printing. Sensors Actuators B Chem..

[37] Li F., Macdonald N.P., Guijt R.M., Breadmore M.C. (2019). Increasing the functionalities of 3D printed microchemical devices by single material, multimaterial, and print-pause-print 3D printing. Lab Chip.

[38] Tan H.W., Tran T., Chua C.K. (2016). A review of printed passive electronic components through fully additive manufacturing methods. Virtual Phys. Prototyp..

[39] Ethan B. (2018). Secor Principles of Aerosol Jet Printing. Flex. Printed Electron..

[40] Oelßner W., Berthold F., Guth U. (2006). The iR drop—Well-known but often underestimated in electrochemical polarization measurements and corrosion testing. Mater. Corros..

[41] Zhmud B., Prof A. (2014). Lube-Tech093-ViscosityBlendingEquations. Lube Mag..

[42] Norland Optical Adhesive 81 Technical Datasheet. https://www.norlandprod.com/adhesives/NOA%2081.html.

[43] Wägli P., Homsy A., De Rooij N.F. (2011). Norland optical adhesive (NOA81) microchannels with adjustable wetting behavior and high chemical resistance against a range of mid-infrared-transparent organic solvents. Sensors Actuators B Chem..

[44] Bartolo D., Degré G., Nghe P., Studer V. (2008). Microfluidic stickers. Lab Chip.

[45] Morel M., Bartolo D., Galas J.C., Dahan M., Studer V. (2009). Microfluidic stickers for cell- and tissue-based assays in microchannels. Lab Chip.

[46] Sollier E., Murray C., Maoddi P., Di Carlo D. (2011). Rapid prototyping polymers for microfluidic devices and high pressure injections. Lab Chip.

[47] Akhatov I.S., Hoey J.M., Swenson O.F., Schulz D.L. (2008). Aerosol focusing in micro-capillaries: Theory and experiment. J. Aerosol Sci..

[48] Binder S., Glatthaar M., Rädlein E., Binder S., Glatthaar M., Edda R. (2014). Analytical Investigation of Aerosol Jet Printing. Aerosol Sci. Technol..

[49] Radhi M.M., Jaffar Al-Mulla E.A., Tan W.T. (2014). Electrochemical characterization of the redox couple of Fe(III)/Fe(II) mediated by grafted polymer electrode. Res. Chem. Intermed..

[50] Gowda J.I., Nandibewoor S.T. (2014). Electrochemical behavior of paclitaxel and its determination at glassy carbon electrode. Asian J. Pharm. Sci..

[51] Brownson D.A.C., Banks C.E. (2014). Interpreting Electrochemistry. The Handbook of Graphene Electrochemistry.

[52] Guemes M., Rahman S.A., Hussain K. (2016). What is a normal blood glucose?. Arch. Dis. Child..

[53] Biscay J., Rama E.C., García M.B.G., Carrazón J.M.P., García A.C. (2011). Enzymatic sensor using mediator-screen-printed carbon electrodes. Electroanalysis.

